# Effect of visual stimulation on epilepsy susceptibility in neonatal hypoglycemic brain injury rats during development

**DOI:** 10.3389/fneur.2025.1587200

**Published:** 2025-06-16

**Authors:** Yifan Sun, Xiao Li, Yan Dong, Xiaoli Zhang, Ling Gan, Tianming Jia

**Affiliations:** ^1^Department of Children's Development and Behavior Center, The Third Affiliated Hospital of Zhengzhou University, Zhengzhou, Henan, China; ^2^Department of Pediatric Neurology, The Third Affiliated Hospital of Zhengzhou University, Zhengzhou, Henan, China

**Keywords:** visual stimulation, neonatal hypoglycemic brain injury, epilepsy susceptibility, BDNF, SYN

## Abstract

**Objective:**

To investigate the effect of visual stimulation on epilepsy susceptibility in neonatal hypoglycemic brain injury (HBIN) rats and its underlying mechanisms.

**Methods:**

Seventy-five 2-day-old Sprague–Dawley rats were divided into three groups: control (N, *N* = 25), model (NH, *N* = 25), and visual stimulation (NH-V, *N* = 25). The NH and NH-V groups were injected with insulin (40 U/kg) on postnatal days 2, 4, and 6, and blood glucose was monitored. The NH-V group received daily 2-h visual stimulation from P14 to P28. At P21, brain Magnetic Resonance Imaging (MRI) was performed. Pentylenetetrazol (PTZ) was injected to induce seizures and recorded at P28. Brain tissue was analyzed for Brain-Derived Neurotrophic Factor (BDNF) and Synaptophysin (SYN) expression.

**Results:**

(i) In the NH and NH-V groups, blood glucose decreased after insulin injection, with behavioral changes observed at 1–4 h. One rat in the NH group had spontaneous seizures. (ii) MRI at 15 days showed occipital lobe abnormalities in 50% of NH rats, with no changes in controls. (iii) In PTZ-induced seizures, the N group had significantly lower seizure scores than the NH group, with the NH-V group showing further reduction. (iv) BDNF and SYN expression were higher in the NH-V group compared to the NH group.

**Conclusion:**

Visual stimulation reduces epilepsy susceptibility in neonatal HBIN rats, likely through upregulation of BDNF and SYN expression in the occipital cortex.

## 1 Background

Neonatal hypoglycemia (NH) is the most common metabolic disorder during the neonatal period. Persistent or recurrent hypoglycemia can lead to hypoglycemic brain injury (HBIN) in neonates (1, 2). HBIN is a prevalent type of perinatal brain injury, characterized by a specific pattern of occipital lobe damage. Numerous clinical studies have demonstrated its imaging features, including early occipital edema followed by liquefactive necrosis (3). Perinatal hypoglycemia is considered a major cause of parieto-occipital brain injury, accounting for 71.4% of cases (4). Recent research has revealed that HBIN is a common cause of symptomatic epilepsy in infants (5, 6). A clinical analysis of 170 cases of epilepsy secondary to HBIN indicated that 96.5% of affected children exhibited gliosis in the occipital lobe, with or without involvement of the parietal lobe (7). Furthermore, more than 80% of children with perinatal occipital encephalomalacia develop secondary epilepsy (4, 8).

The occipital lobe, serving as the visual processing center, is a critical area for early neurodevelopment during infancy. Visual information constitutes over 80% of sensory input to the human brain, and postmortem studies of human brains have shown that the developmental peak of the visual cortex occurs at around 4 months of age (9). Notably, the average onset age of infantile spasms secondary to HBIN is between 4 and 6 months, suggesting a potential temporal association between the two conditions. Current studies indicate that epilepsy secondary to HBIN is strongly associated with occipital lobe damage. However, the impact of visual stimulation on seizure activity in the context of injured occipital lobes has yet to be explored.

This study established a rat model of HBIN with occipital lobe injury to investigate the effect of visual stimulation. Subthreshold doses of pentylenetetrazol (PTZ) were used to induce seizures, and seizure scores were recorded to compare differences in epilepsy susceptibility between groups. Additionally, immunohistochemical analysis was performed to measure the expression levels of brain-derived neurotrophic factor (BDNF) and synaptophysin (SYN) in the occipital cortex, providing preliminary insights into the potential mechanisms by which visual stimulation influences epilepsy susceptibility in HBIN.

## 2 Materials and methods

### 2.1 Experimental animals and grouping

Seventy-five 2-day-old, SPF-grade healthy Sprague–Dawley (SD) rats (weight: 5.23–8.28 g, purchased from the Henan Experimental Animal Center, Animal License Number: SCXK (Yu) 2017–0001) were used in this study. Rats were housed under 12/12-h light/dark cycle in the animal facility, with access to food and water ad libitum. Rats were randomly divided into three groups: Normal Control (N, *n* = 25), Neonatal Hypoglycemia (NH, *n* = 25), and Neonatal Hypoglycemia with Visual Stimulation (NH-V, *n* = 25). Ethical approval was granted from local ethics committee.

### 2.2 Model preparation

Rats in the NH and NH-V groups were intraperitoneally injected with insulin (40 U/kg, 10 mL/400 U, Jiangsu Wanbang) on postnatal days 2, 4, and 6 to induce hypoglycemia. Blood glucose levels were measured with blood glucose meter (Accu-Chek Guide Link [Model: 114], Roche) by heel blood sampling before injection, and 1, 2, 3, and 4 h after injection. After the 5th blood glucose measurement, a 2 mL/kg intraperitoneal injection of 50% glucose was administered to terminate hypoglycemia, and the rats were returned to their mothers. Rats in the N group received an equivalent volume of saline injections at the same time points, with blood glucose monitored in the same manner, and were also returned to their mothers. Use random sampling to select 5 rats from both the NH group and the NHV group, and 10 newborn rats from the N group for comparative analysis of blood glucose levels after insulin injection. Rats in the NH and NH-V groups were included in the study if their blood glucose levels reached ≤0.8 mmol/L 4 h after insulin injection, and they exhibited hypoglycemic symptoms such as drowsiness and decreased skin temperature. Rats that experienced spontaneous seizures or death during the model development process were excluded from the study.

### 2.3 Design and intervention of visual stimulation

After eye opening (P14), rats in the NH-V group were subjected to visual stimulation for 2 h per day, in sync with the sleep schedule of infants in China, with the intervention lasting for 14 consecutive days. A tablet computer was placed on both sides of the cage wall (The distance between tablet computer and the wall is 2 cm), playing chapters 1–8 of “Dragon Warrior” (a regular TV series with rich color and graphic variations) in silent mode. The brightest stripe in the chapters exceeds 50 cd/m^2^ (appearing for more than 2 min); within the chapters, there are frequent transitions between bright and dark scenes, with a brightness difference between darker and brighter images reaching 30 cd/m^2^; saturated red images are present in the scenes; the chapters contains numerous flickering scenes, with the flickering area covering more than a quarter of the screen, a high flickering frequency, and more than 3 flickers within a second. The stimulation was given at fixed times: 9:00–10:00 AM and 2:00–3:00 PM. The rats were allowed to move freely during the intervention, ensuring effective exposure to the stimulus. To avoid stress interference during handling, rats in the N and NH groups were removed to the cage with general environmental changes at the same times, with no visual stimulus, serving as the control.

### 2.4 Magnetic resonance imaging (MRI) detection

Use random sampling to select 10 newborn rats from both the N group and the NH group. Rats were anesthetized with isoflurane (550 ml/min, 3 min) and underwent head axial scans. Magnetic Resonance Imaging (MRI) system (4.7 T, MR Solution, Model: MRS-4717). The scan parameters were as follows: T2-weighted imaging (T2W), TE/TR: 51/5000 ms, Field of View (FOV): 25, slice thickness/inter-slice distance: 1.5/0.1 mm, imaging software version: PreclinicalScan 1.2.

### 2.5 Seizure induction

On day 14 post-modeling (P28), rats from each group were intraperitoneally injected with subthreshold doses of PTZ (50 mg/kg, 98%, 1 g, Saikelin) (10). Seizure activity was observed for 10 min and seizure scores were recorded. Seizures were classified and scored according to the following scale (adapted from Baran H, 1994): no seizure = 0 points; eye closing, whisker twitching, sneezing, facial myoclonus, or staring = 1 point; head nodding with severe facial myoclonus = 2 points; unilateral or bilateral forelimb myoclonus = 3 points; standing with bilateral forelimb myoclonus = 4 points; standing with postural imbalance, falling, and generalized clonic seizures = 5 points; persistent generalized clonic seizures = 6 points (11).

### 2.6 Sample collection and detection

In accordance with the “Guidelines on the Humane Treatment of Laboratory Animals” issued by the Ministry of Science and Technology of China, rats are euthanized using the carbon dioxide inhalation method, which is simple, safe, effective, inexpensive, and does not contaminate tissues or the environment. The procedure involves placing the rats in a carbon dioxide anesthesia chamber, opening the carbon dioxide gas valve, and waiting for the animals to gradually lose consciousness. The carbon dioxide concentration is then increased to 100%, rendering the animals unconscious, with no pinch reflex or loss of muscle tone or corneal reflex (applicable only to larger animals and rabbits). The animals are then ventilated for an additional 2 min to ensure death.

After euthanizing the rats, whole brain tissue was immediately removed and fixed. The fixed brain tissues were embedded in paraffin (Cas No.: 8002-74-2, Sigma-Aldrich), and consecutive coronal slices of the occipital lobe (thickness: 4 μm) were prepared with paraffin microtome (CUT 6062, SLEE). Immunohistochemical staining was used to detect the expression of BDNF and SYN. Antigen retrieval was performed, followed by serum blocking (40 min), incubation sequentially with primary and secondary antibodies at 37°C for 20 min. Color development was achieved using aminoethylcarbazole, and hematoxylin counterstaining was performed. After dehydration, clearing, and mounting, sections were examined with Image-Pro Plus (Media Cybernetics) under a high-power microscope (Digital Microscope, VHX-X1, Keyence). The expression intensity of positive signals was assessed by calculating the mean optical density (IOD/positive area) of the images. Antibodies include: rabbit anti-BDNF antibody (50 μL, Affinity, Catalog No.: DF-6387); mouse anti-SYN antibody (100 μL, Servicebio, Catalog No.: GB12553); Anti-Rabbit IgG(H + L) (100 μL, ABclonal, Catalog No.: AS070); Anti-mouse IgG(H + L) (100 μL, abcam, Catalog No.: ab150113).

### 2.7 Statistical analysis

Data analysis was performed using SPSS 22.0 software. Quantitative data are presented as mean ± standard deviation (±s). Independent sample *t*-tests were used for inter-group comparisons, while one-way repeated measures ANOVA and paired *t*-tests were applied for intra-group comparisons. A *p*-value of <0.05 was considered statistically significant, and all analyses were two-sided.

## 3 Results

### 3.1 Model preparation

To balance the sample sizes between the NH + NHV group (50 rats) and the N group (25 rats) for comparative analysis, a random sampling method was employed to select 5 rats from each of the NH and NHV groups, and 10 rats from the N group for analysis. Blood glucose levels of neonatal rats before insulin administration on postnatal days 2, 4, and 6 (4.302 ± 0.350, 5.120 ± 0.350, 5.360 ± 0.690) were not significantly different from those of the saline control group (4.310 ± 0.410, 5.060 ± 0.490, 5.370 ± 0.450) (*p* = 0.954, *p* = 0.754, *p* = 0.754).

In the NH and NH-V groups, blood glucose levels significantly decreased 1 h after insulin injection on postnatal days 2, 4, and 6 (*p* = 0.001, *p* = 0.001, *p* = 0.001). Blood glucose levels decreased further after 2 h compared to 1 h after injection (*p* = 0.001, *p* = 0.001, *p* = 0.042). Data from 3 and 4 h after insulin injection were below the measurement threshold (<0.06 mmol/L). Data were calculated as half of the minimum threshold, and the result is still below the threshold. So statistically analyzed was not conducted.

In the N group, blood glucose levels on postnatal days 2, 4, and 6 were analyzed by one-way repeated measures analysis of variance (F: 0.141, 1.429, 0.837; P: 0.914, 0.261, 0.468), showing no statistically significant differences in blood glucose levels before and 4 h after insulin injection (*p* > 0.050), as shown in Table 1.

**Table 1:** Blood glucose after insulin injection mmol/L (±s).

Groups		Number	Blood glucose		
			2d	4d	6d
NH group+ NH-V group		10			
	Before injection		4.320 ± 0.350^c^	5.120 ± 0.350^d^	5.360 ± 0.690^e^
	1 h after injection		1.650 ± 0.220^*^	1.670 ± 0.180^*^	1.780 ± 0.300^*^
	2 h after injection		0.960 ± 0.150^#^	1.150 ± 0.230^#^	1.270 ± 0.390^#^
	3 h after injection^a^		≤0.900	≦1.000	≤1.200
	4 h after injection^a^		≦0.600	≦0.600	≦0.800
N group		10			
	Before injection		4.310 ± 0.410	5.060 ± 0.490	5.370 ± 0.450
	1 h after injection		4.430 ± 0.610	4.660 ± 0.600	4.940 ± 0.590
	2 h after injection		4.290 ± 0.470	5.020 ± 0.400	5.230 ± 0.450
	3 h after injection		4.330 ± 0.510	5.140 ± 0.650	5.100 ± 0.720
	4 h after injection		4.300 ± 0.510	4.950 ± 0.360	5.170 ± 0.610
	F^b^		0.141	1.429	0.837
	*P* ^b^		0.914	0.261	0.468

a: Because some rats’ blood glucose is lower than the lower measurement limit of the blood glucose meter (0.600 mmol/L), no statistical processing; b: Comparison of control groups; c, d, e: 2-4dpre - injection comparison between the experimental and control groups (tc = 0.059, Pc = 0.954; td = 0.318, Pd = 0.754; te = 0.038, Pe = 0.754); *: Comparison before injection; #: comparison after injection 1 h.

### 3.2 MRI results

**Figure 1:**
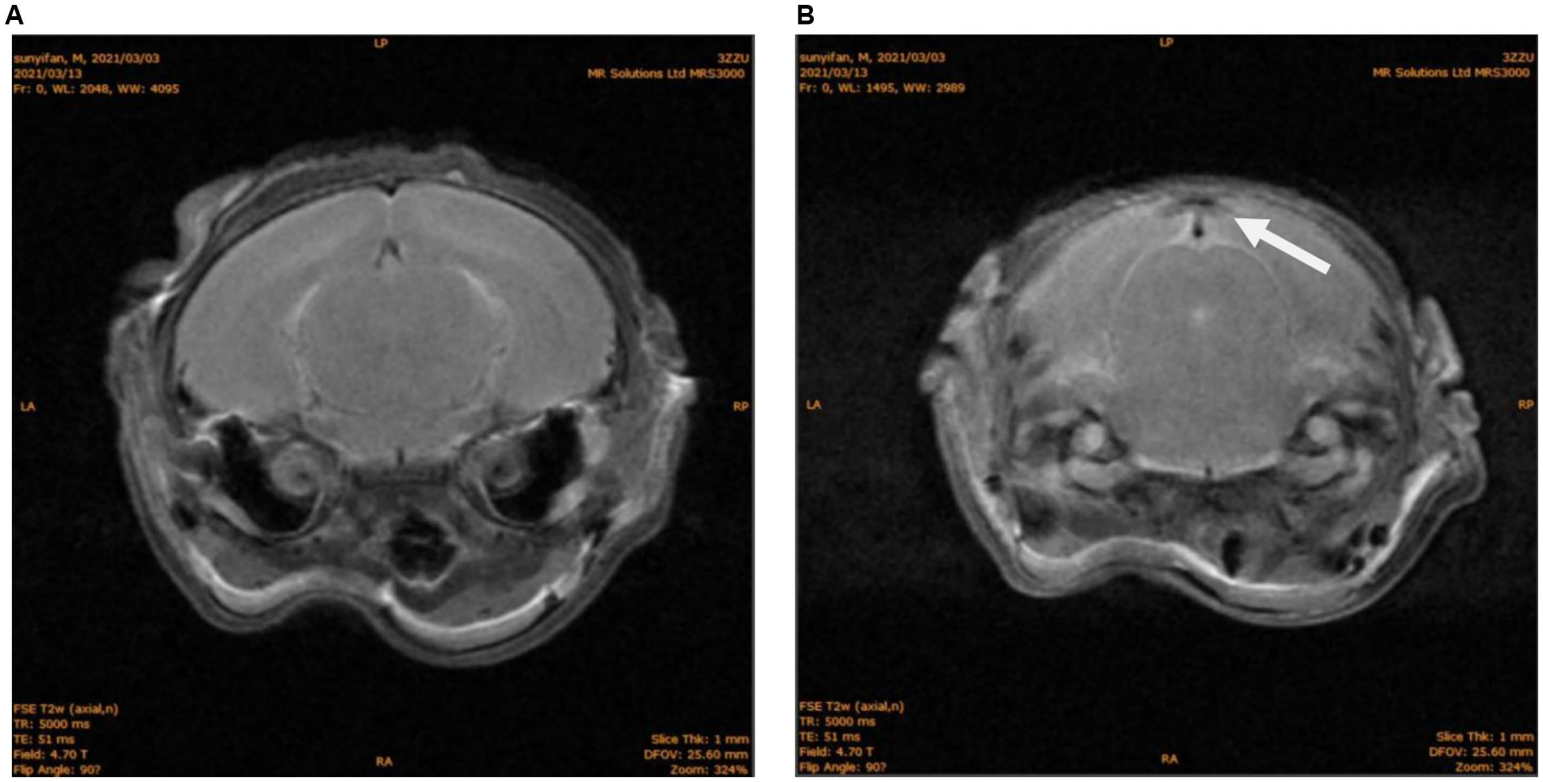
MRI of brain structures in neonatal rats. **(A)** N group T2W phase, No abnormal signal. **(B)** NH group T2W phase, abnormal density of occipital lobe, as shown with white arrow.

Fifteen days post-modeling, MRI of the N group (*n* = 10) showed symmetrical and intact brain structure, with uniform density of both white matter and gray matter (Figure 1A). In the NH group, 5 rats exhibited structural damage of varying severity, characterized by mixed signals in the occipital region, predominantly low signals (Figure 1B). The remaining 5 rats showed no significant abnormalities.

### 3.3 Seizure induction results

Fourteen days post-modeling, seizure induction results showed that the N group had significantly lower seizure scores compared to the NH group (*t* = −3.431, *p* = 0.008). The NH-V group also had lower seizure scores compared to the NH group, with a statistically significant difference (*t* = −2.283, *p* = 0.048), as shown in Table 2.

**Table 2:** P28 PTZ induced seizure score of each group ( $\bar{x}$ ± s, *n* = 25).

Groups	*n*	Scores
N	25	2.400 ± 0.843
NH	25	4.100 ± 0.876^a^
NH-V	25	3.000 ± 1.155^b^
F		10.689
P		<0.001

^b^*P* < 0.050. N: normal control; NH: Neonatal Hypoglycemia; NH-V: Neonatal Hypoglycemia- Visual stimuli; compared with N group, ^a^*P* < 0.050; compared with NH group, b*P* < 0.050.

### 3.4 Expression of BDNF and SYN in the occipital cortex

**Figure 2:**
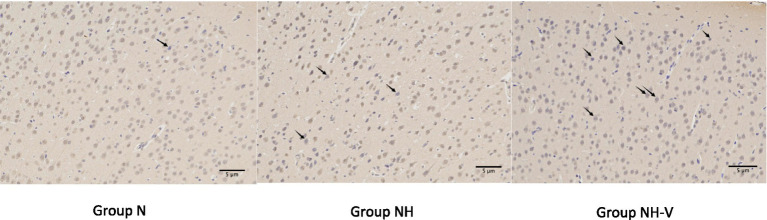
The expressions of BDNF in occipital cortex of rats under light microscope, in each group. Representative immunohistochemical expression of Brain-Derived Neurotrophic Factor (BDNF) in occipital cortex of rats under light microscope (x200) in control (N), model (NH) and visual-stimulation (NH-V) groups. Black arrows indicate the BDNF expression.

**Figure 3:**
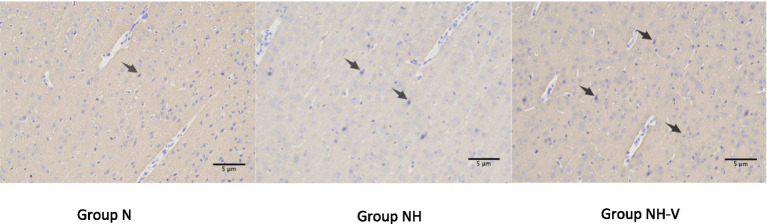
The expressions of SYN in occipital cortex of rats under light microscope in each group. Representative immunohistochemical expression of Synaptophysin (SYN) in occipital cortex of rats under light microscope (x200) in control (N), model (NH) and visual-stimulation (NH-V) groups. Black arrows indicate the SYN expression.

Compared to the N group, the NH group showed an increase in BDNF expression in the occipital cortex (*p* = 0.010). Additionally, the NH-V group had higher BDNF expression in the occipital cortex compared to the NH group (*p* = 0.025), as shown in Table 3 and Figure 2 (Mark with black arrow). Furthermore, compared to the NH group, the NH-V group exhibited increased SYN expression in the occipital cortex (*p* = 0.012), as shown in Table 3 and Figure 3 (Mark with black arrow).

**Table 3:** Comparison of BDNF and SYN expression in occipital cortex of rats ( $\bar{x}$ ± s, *n* = 25).

Groups	*n*	BDNF	SYN
N	25	0.034 ± 0.004	0.024 ± 0.003
NH	25	0.040 ± 0.006^a^	0.021 ± 0.002^a^
NH-V	25	0.043 ± 0.006^b^	0.235 ± 0.004^b^
F		10.787	4.590
P		<0.001	0.016

BDNF: Brain-derived nerve growth factor, compared with N group, ^a^*P* < 0.050, compared with NH group, ^b^*P* < 0.050; SYN; Synaptic, compared with NH group, ^b^*P* < 0.05.

## 4 Discussion

Epilepsy is a clinical phenomenon caused by abnormal neuronal discharge in the brain, often accompanied by changes in the synchrony of neural networks (12). Neonatal hypoglycemia (NH) can lead to specific damage in the occipital lobe, resulting in disorganized brain structures and an increased risk of epilepsy (4). The occipital lobe, as the visual center, receives visual stimuli that induce synchronized oscillations in neural networks, enhancing functional cooperation between neurons (13). Previous studies have shown that visual stimuli can trigger gamma oscillations (30–100 Hz) via the retina, which are transmitted to the occipital cortex to synchronize neuronal activity and regulate synchrony between brain regions, facilitating information exchange (14–17). When pathological processes cause a lack of neuronal synchronization, gamma oscillations may exceed the normal physiological range, providing a foundation for epilepsy (18, 19). Based on this theory, we hypothesized that enhanced visual stimuli might increase the susceptibility to epilepsy in HBIN rats, and designed this experiment to test this hypothesis.

This study aims to explore the effects of visual stimulation on epileptic susceptibility in neonatal hypoglycemic brain injury (HBIN) rats and its potential mechanisms. In 2005, American epilepsy experts identified the characteristics of flashing lights that are prone to triggering seizures: a flash brightness exceeding 20 cd/m (where cd is the luminance unit candela), a frequency greater than 3 Hz, with the brightest stripe having a luminance greater than 50 cd/m and a duration exceeding 0.5 s (20). The “Guidelines for Reducing Photosensitive Epileptic Seizures Caused by Television” released by the ITU Radiocommunication Sector in 2020 provides a detailed description of situations that may lead to photosensitive epileptic seizures: Potentially harmful flicker occurs when there is a pair of opposite changes in brightness (i.e., brightness first increases and then decreases, or first decreases and then increases). Potentially harmful flicker sequences occur when the screen brightness of darker images is below 160 cd/m^2^, or when the screen brightness difference between darker and brighter images is 20 cd/m or greater. Regardless of brightness, transitions to or from saturated red may also be harmful. Additionally, the combined area of those simultaneous flickers occupying more than a quarter of the displayed screen area, with more than three flickers occurring in any 1-s period, can also be problematic (21). Due to the simple daily life scenarios of newborns, who typically remain indoors for a month after birth and only begin venturing outdoors after a hundred days in China, the control group did not incorporate color stimulation. Instead, it only presented general environmental changes typical of normal living situations. The results show that visual stimulation significantly reduces epileptic susceptibility in HBIN rats, and this effect may be achieved by upregulating the expression of brain-derived neurotrophic factor (BDNF) and synaptophysin (SYN) in the occipital cortex. First, we successfully established an occipital lobe injury model in HBIN rats. Current neonatal hypoglycemia models mainly include mild repeated hypoglycemia models (22) and severe repeated hypoglycemia models (23), both of which can detect neuronal apoptosis in sensitive areas such as the occipital cortex at the cellular level but show no significant imaging abnormalities. Another commonly used model is the single prolonged hypoglycemia model (24), which induces hypoglycemia by fasting for 2 h and maintaining low blood sugar for 7 h, with a high incidence of acute epilepsy. However, the epileptic manifestations of this model do not accurately reflect the clinical course of secondary epilepsy in infants. Clinical studies show that the degree of brain injury caused by hypoglycemia is closely related to both the severity and duration of hypoglycemia (25). Based on our pre-experiment results, we extended the exposure time to hypoglycemia in this study and successfully established a model suitable for studying secondary epilepsy.

Fifteen days later (equivalent to 3–5 years of age in humans), we randomly selected 10 rats for head MRI scans. Animals raised in the same environment exhibit no significant differences in phenotypic traits such as body weight. Rats are housed five per cage, and two are randomly selected from each cage to avoid large variations in test results. Among them, five rats showed varying degrees of low signal in the occipital lobe on T2-weighted images, consistent with the clinical and animal model findings of occipital lobe damage caused by hypoglycemia (26), providing a reliable basis for the subsequent epilepsy susceptibility experiments.

Most previous studies on the relationship between visual stimuli and epilepsy have focused on fixed-frequency visual stimulation to investigate the effects of specific frequency oscillations on the mechanisms of epilepsy (27). In contrast, this study investigates how daily exposure to visual stimuli during the latent period of epilepsy onset after occipital lobe injury in HBIN rats affects their susceptibility to epilepsy. We chose electronic video stimuli, which are commonly used in basic research on visual system diseases and are part of the everyday environment. These stimuli are characterized by changing shapes, light–dark contrasts, and rich color variations, with an unpredictable frequency. In contrast, the control group rats were only exposed to a relatively simple visual environment in the animal room, which was less diverse in contrast, shape, and color. Therefore, a significant difference was observed between the two groups. However, the results show that the epileptic scores in the NH-V group were significantly lower than in the NH group, indicating that visual stimulation reduced the epileptic susceptibility of HBIN rats, which was different from our initial hypothesis. Possible reasons for this discrepancy include: (1) Gamma oscillations play an important role in information encoding and neural network synchrony (28), and task load can enhance synchrony (29). Although visual stimuli might promote network synchrony, they did not significantly impact epileptic susceptibility; (2) The intensity, pattern, and duration of visual stimulation in this study might have increased susceptibility in some cases. However, previous studies suggest that enriched environments promote neural repair after brain injury (30), and visual stimulation, as part of such an enriched environment, may reduce epileptic susceptibility by facilitating brain function recovery.

Regarding the preparation of the neonatal hypoglycemia rat model, we combined indicators such as accelerated respiration and heart rate, decreased skin temperature, and changes in behavior like apathy or somnolence to determine the success of the model (31). To further verify the hypoglycemic condition, 10 rats were randomly selected from both the experimental group and the control group for blood glucose measurement, followed by insulin injection. In the hypoglycemia model, insulin injection significantly reduced blood glucose levels in the NH and NH-V groups, showing typical symptoms of hypoglycemia. No changes in blood glucose were observed in the N group, indicating that saline injection did not interfere with normal physiological functions. Head MRI scans showed low signals in the occipital lobe of NH group rats, indicating occipital lobe damage, which is inconsistent with previous studies on hypoglycemic brain injury (3, 4).

In the epilepsy susceptibility test, the PTZ-induced epilepsy scores showed that the severity of seizures in the NH group was significantly higher than in the N group, while the NH-V group had lower scores than the NH group. This indicates that visual stimulation can significantly alleviate the severity of seizures in HBIN rats, consistent with previous studies on environmental factors or behavioral interventions influencing epilepsy (5, 6). Visual stimulation, as an external sensory input, may enhance the neural adaptability and cortical plasticity of rats, potentially counteracting the epileptic susceptibility caused by HBIN.

Further immunohistochemical analysis showed that the expression of BDNF in the occipital cortex of NH group rats was significantly higher than that of the N group, and the NH-V group exhibited even higher BDNF expression, which was positively correlated with the changes in SYN expression. This suggests that visual stimulation not only increases BDNF expression but may also enhance the neuroprotective effect by promoting the expression of SYN, which is associated with synaptic plasticity. BDNF, an important neurotrophic factor, has been shown to have repair and regenerative effects following neural injury (7), while SYN is a key protein involved in synaptic function and the formation of synapses (8). Therefore, visual stimulation may improve neurological function and reduce epileptic susceptibility in HBIN rats by upregulating the expression of BDNF and SYN.

Furthermore, BDNF has been proven to promote neural repair and regeneration after brain injury (32), and its expression is upregulated in various types of brain injuries (33). In this study, both NH-V and NH groups exhibited increased BDNF expression in the occipital cortex, with enriched visual stimulation further enhancing BDNF expression (34). Previous research indicates that the BDNF/TrkB pathway helps prevent or suppress epilepsy in brain injury models (35), which is consistent with the lower epileptic susceptibility observed in the NH-V group. BDNF, an important molecule for synaptic plasticity, is closely related to synaptic remodeling and activity (36). After brain injury, BDNF promotes compensatory neural network remodeling (37). SYN, a presynaptic membrane protein widely distributed in nerve terminals, participates in synaptic structural and functional changes and is closely related to BDNF in synaptic remodeling (38). This study indicates that visual stimulation may reduce epileptic susceptibility by promoting synaptic remodeling in damaged areas, which could be one of the mechanisms through which visual stimulation decreases epileptic susceptibility in developmental HBIN rats (39–41).

However, there are some limitations to this study. The animals used in this experiment were 2-day-old rats, where inhibitory synapses had not yet matured. Cortical neurons in the visual cortex exhibited stronger adaptability to continuous flash stimulation compared to adult rats. Additionally, since excitatory synapses in the cortex are regulated by inhibitory synapses, the immature inhibitory system in young rats might lead to enhanced short-term depression of excitatory synapses and increased frequency-dependent adaptability to flash stimulation. Therefore, this study employed a stronger flash stimulus. However, further research is still needed to corroborate these findings. Second, while visual stimulation has a significant effect on reducing epileptic susceptibility, the exact neural mechanisms remain unclear. Future studies should explore the effects of visual stimulation on neural circuit remodeling, neuronal activity patterns, and different neurotransmitter systems. Additionally, although we analyzed BDNF and SYN expression through immunohistochemistry, we did not investigate other molecular mechanisms related to epileptic susceptibility, such as neuroinflammation and ion channel changes. Overall, this study provides experimental evidence for the impact of visual stimulation on epileptic susceptibility in neonatal hypoglycemic brain injury rats and offers preliminary insights into the role of BDNF and SYN in this process. Future research can further explore the mechanisms of visual stimulation and evaluate its potential clinical applications for hypoglycemic brain injury and epilepsy treatment.

## Data Availability

The raw data supporting the conclusions of this article will be made available by the authors, without undue reservation.
